# Supplementary material for: Coinfection of Cage-Cultured Spotted Sea Bass (Lateolabrax maculatus) with Vibrio harveyi and Photobacterium damselae subsp. piscicida Associated with Skin Ulcer
Source: Microorganisms. 2024 Feb 29;12(3):503. doi: 10.3390/microorganisms12030503 (PMC10975084; doi:10.3390/microorganisms12030503)

Supplementary data

Table S1. Biochemical characteristics of *Vibrio harveyi* NH-LM1^T and *Photobacterium damselae* subsp. *piscicida* NH-LM2^T.

Characteristic	NH-LM1^T	NH-LM2^T
**Utilize of**		
Potassium nitrate	-	-
L-tryptophan	+	-
Dextrose	+	+
L-Arginine	+	+
Urea	+	+
Aescin iron citrate	+	+
Gel (bovine source)	+	-
4-Nitrobenzene-*β*-D-galactopyranoside	+	+
Dextrose	-	-
L-arabinose	-	-
D-mannose	-	-
D-mannitol	-	-
N-Acetyl Glucosamine	-	-
D-maltose	-	-
Potassium Gluconate	-	-
Capric acid	-	-
Adipic acid	-	-
Malic acid	-	+
Sodium citrate	-	-
Phenylacetic acid	-	-
**Enzyme activity**		
Alkaline phosphatase	+	+
Esterase(C4)	+	+
Esterase lipase (C8)	+	+
Lipase (C14)	+	+
Leucine arylamidase	+	+
Valine arylamidase	+	+
Cystine arylamidase	+	+
Trypsin	+	-
Chymotrypsin	+	-
Acid phosphatase	+	+
Naphthol-AS-BI-phosphohydrolase	+	+
α-galactosidase	-	-
β-galactosidase	-	-
β-uronidase	-	-
α-glucosidase	+	-
β-glucosidase	-	-
N-acetyl-*β*-glucosaminidase	+	-
α-mannosidase	-	-
α-fucosidase	-	-
Oxidase	+	+
Catalase	+	+

+, positive; –, negative.

Table S2 Clusters of Orthologous Group (COG) annotations of strain NH-LM1 genome.

**Categories**	**Function**	**Gene number**	**Ratio (%)**
C	RNA processing and modification	1	0.03
D	Energy production and conversion	221	6.48
E	Cell cycle control, cell division, chromosome partitioning	61	1.79
F	Amino acid transport and metabolism	284	8.33
G	Nucleotide transport and metabolism	107	3.14
H	Carbohydrate transport and metabolism	190	5.58
I	Coenzyme transport and metabolism	199	5.84
J	Lipid transport and metabolism	114	3.35
K	Translation, ribosomal structure and biogenesis	259	7.60
L	Transcription	245	7.19
M	Replication, recombination and repair	149	4.37
N	Cell wall/membrane/envelope biogenesis	232	6.81
O	Cell motility	88	2.58
P	Posttranslational modification, protein turnover, chaperones	183	5.37
Q	Inorganic ion transport and metabolism	180	5.28
R	Secondary metabolites biosynthesis, transport and catabolism	38	1.12
S	General function prediction only	217	6.37
T	Function unknown	134	3.93
U	Signal transduction mechanisms	207	6.07
V	Intracellular trafficking, secretion, and vesicular transport	100	2.93
W	Defense mechanisms	111	3.26
Z	Extracellular structures	41	1.20

Table S3 Clusters of Orthologous Group (COG) annotations of strain NH-LM2 genome.

**Categories**	**Function**	**Gene number**	**Ratio (%)**
C	RNA processing and modification	1	0.02
D	Energy production and conversion	263	5.76
E	Cell cycle control, cell division, chromosome partitioning	71	1.55
F	Amino acid transport and metabolism	379	8.30
G	Nucleotide transport and metabolism	110	2.41
H	Carbohydrate transport and metabolism	278	6.09
I	Coenzyme transport and metabolism	225	4.93
J	Lipid transport and metabolism	168	3.68
K	Translation, ribosomal structure and biogenesis	272	5.96
L	Transcription	364	7.97
M	Replication, recombination and repair	184	4.03
N	Cell wall/membrane/envelope biogenesis	298	6.53
O	Cell motility	129	2.83
P	Posttranslational modification, protein turnover, chaperones	223	4.88
Q	Inorganic ion transport and metabolism	242	5.30
R	Secondary metabolites biosynthesis, transport and catabolism	64	1.40
S	General function prediction only	312	6.83
T	Function unknown	184	4.03
U	Signal transduction mechanisms	352	7.71
V	Intracellular trafficking, secretion, and vesicular transport	167	3.66
W	Defense mechanisms	154	3.37
Z	Extracellular structures	70	1.53

Figures S1. Circular maps of the six plasmids of *Vibrio harveyi* NH-LM1. The base pairs are indicated the inside circle (Circle 1). Circle 1 represents the coding genes colored according to their functional annotations based on COG database. Circle 2, GC content; Circle 3, GC skew+ (green) and GC skew- (purple). (A-F) Plasmid 1 to 6.

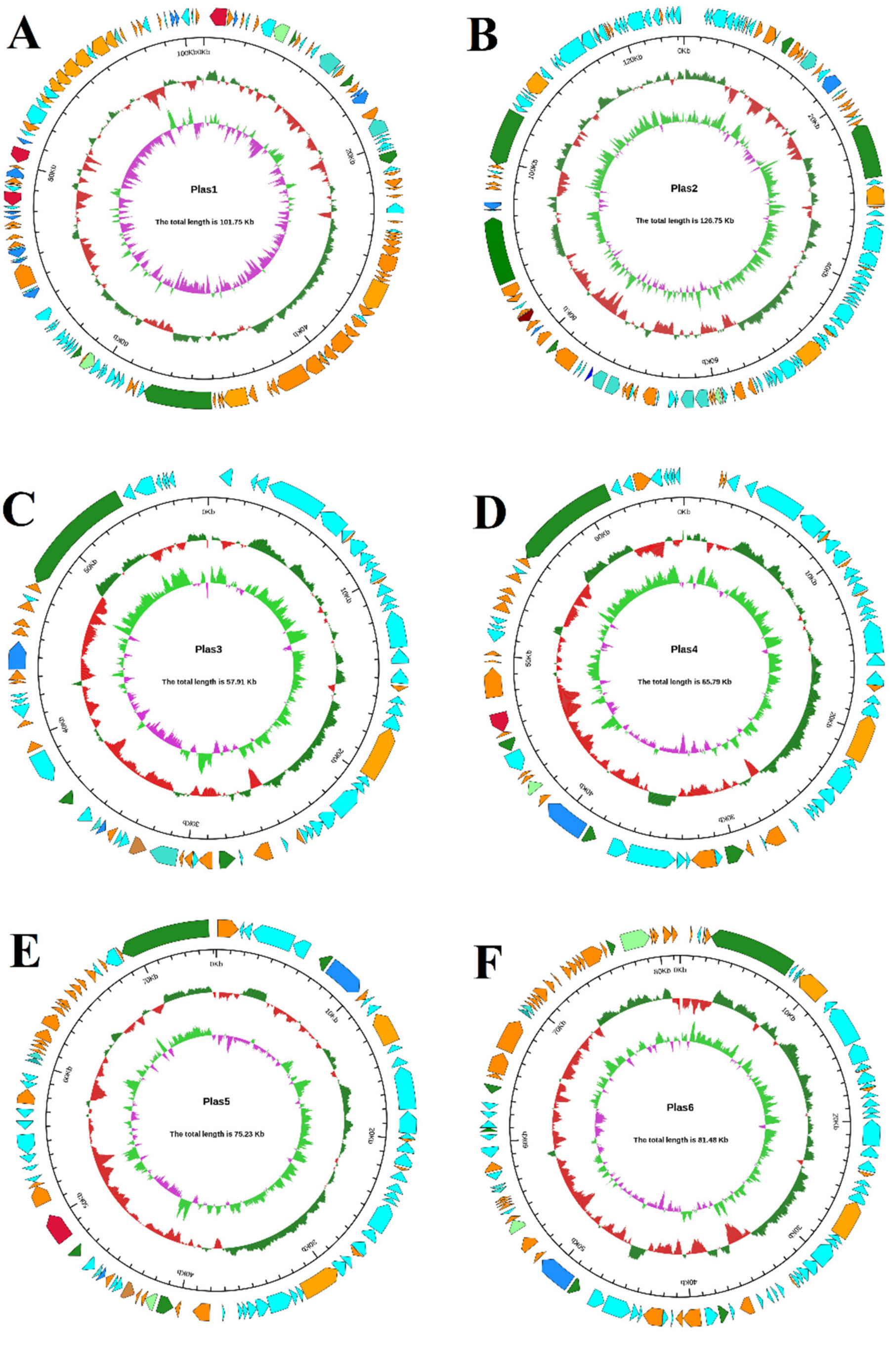

Figure S2. Circular maps of the three plasmids of *Photobacterium damselae* subsp. *piscicida* NH-LM2. The base pairs are indicated the inside circle (Circle 1). Circle 1 represents the coding genes colored according to their functional annotations based on COG database. Circle 2, GC content; Circle 3, GC skew+ (green) and GC skew- (purple). (A-C) Plasmid 1 to 3.

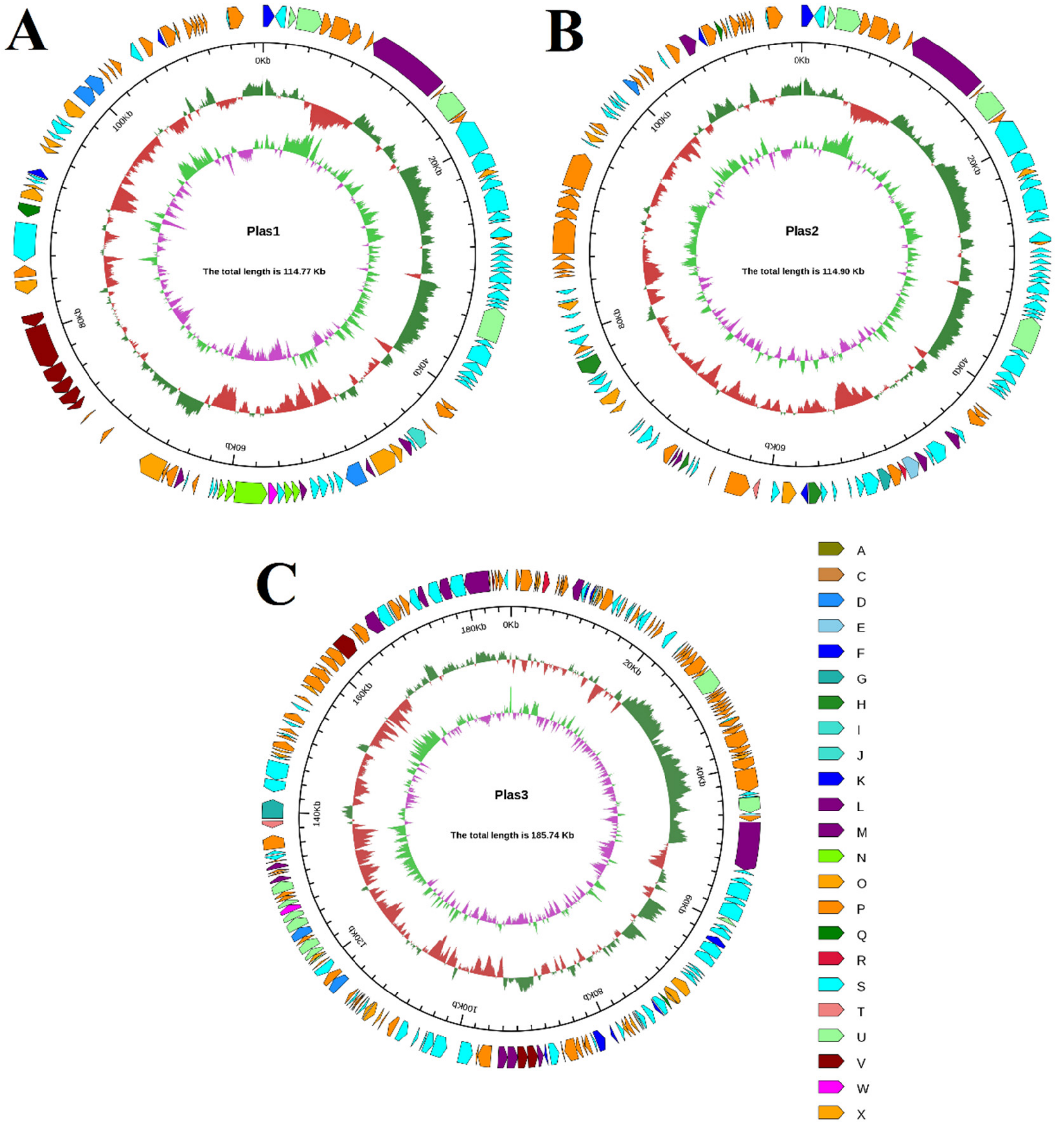

Supplement: Supplementary file 1 [file microorganisms-12-00503-s001.zip › microorganisms-2878953-supplementary.pdf]
